# Circulating miR-200 family as predictive markers during systemic therapy of metastatic breast cancer

**DOI:** 10.1007/s00404-022-06442-2

**Published:** 2022-03-02

**Authors:** Chiara Fischer, Thomas M. Deutsch, Manuel Feisst, Nathalie Rippinger, Fabian Riedel, Andreas D. Hartkopf, Sara Y. Brucker, Christoph Domschke, Carlo Fremd, Laura Michel, Barbara Burwinkel, Andreas Schneeweiss, Andrey Turchinovich, Markus Wallwiener

**Affiliations:** 1 Department of Obstetrics and Gynecology, University of Heidelberg, Im Neuenheimer Feld 440, 69120 Heidelberg, Germany; 2 Institute of Medical Biometry, University of Heidelberg, Im Neuenheimer Feld 130.3, 69120 Heidelberg, Germany; 3 Department of Obstetrics and Gynecology, University of Tübingen, Calwerstraße 7, 72076 Tübingen, Germany; 4 National Center for Tumor Diseases, Im Neuenheimer Feld 460, 69120 Heidelberg, Germany; 5 Molecular Epidemiology C080, German Cancer Research Center (DKFZ), Im Neuenheimer Feld 581, 69120 Heidelberg, Germany

**Keywords:** Metastatic breast cancer, Liquid biopsy, Circulating microRNAs, miR-200 family, Survival

## Abstract

**Purpose:**

Circulating miRNAs can provide valid prognostic and predictive information for breast cancer diagnosis and subsequent management. They may comprise quintessential biomarkers that can be obtained minimally invasively from liquid biopsy in metastatic breast cancer patients. Therefore, they would be clinically crucial for monitoring therapy response, with the goal of detecting early relapse. This study investigated miRNA expression in patients with early and/or late relapse, and the predictive value for assessing overall (OS) and progression-free survival (PFS).

**Methods:**

Forty-seven patients with metastatic breast cancer from the University Women’s Hospital Heidelberg were enrolled in this study. Expression of miR-200a, miR-200b, miR-200c, miR-141, and miR-429 was analyzed by RT-qPCR before a new line of systemic therapy and after the first cycle of a respective therapy. Tumor response was assessed every 3 months using the RECIST criteria. Statistical analysis focused on the relation of miR-200s expression and early vs. late cancer relapse in relation to systemic treatment. The association of miRNAs with PFS and OS was investigated.

**Results:**

Before starting a new line of systemic therapy, miR-429 (*p* = 0.024) expression was significantly higher in patients with early relapse (PFS ≤ 4 months) than in patients with late relapse (PFS > 4 months). After one cycle of systemic therapy, miR-200a (*p* = 0.039), miR-200b (*p* = 0.003), miR-141 (*p* = 0.017), and miR-429 (*p* = 0.010) expression was higher in early than in late progressive cancer. In addition, 4 out of 5 miR-200 family members (miR-200a, miR-200b, miR-141, and miR-429) predicted PFS (*p* = 0.048, *p* = 0.008, *p* = 0.026, and *p* = 0.016, respectively). Patients with heightened miRNA levels showed a significant reduction in OS and PFS.

**Conclusion:**

Circulating miR-200s were differentially expressed among patients with late and/or early relapse. 4 of 5 members of the miR-200 family predicted significantly early relapse after systemic treatment. Our results encourage the use of circulating miR-200s as valuable prognostic biomarkers during metastatic breast cancer therapy.

**Supplementary Information:**

The online version contains supplementary material available at 10.1007/s00404-022-06442-2.

## Introduction

Breast cancer is the most frequently diagnosed cancer entity worldwide, with about 2.1 million new cases having been predicted for 2018 a quarter (24.2%) of all cancer cases among women [1]. In 2018, Germany anticipated 71,900 new cases of breast cancer and a total of 18,136 breast cancer-related deaths [2].

Metastatic breast cancer constitutes a palliative scenario requiring an entirely different treatment approach that meticulously focuses on symptom management and improving patients’ quality of life as opposed to measures for prolonging life. Thus, it is of utmost importance to provide treatment according to a patient’s stage of disease and the previously specified prognosis, thereby preventing unnecessary suffering due to inappropriate therapeutic measures and/or lack of treatment. Here, biomarkers are required that can be easily obtained and reliably reflect the current tumor burden and prognosis.

Current research emphasizes the role of readily obtainable tumor-derived blood components that have predictive and prognostic value. Such components include circulating microRNA (miRNA), circulating tumor cells, cell-free DNA, circulating tumor DNA, circulating tumor RNA, exosome vesicles, and tumor-educated platelets [3].

miRNAs constitute small noncoding RNA molecules that regulate the expression of multiple genes by initiating translational silencing and/or degrading their respective cognate mRNA targets [4]. The miR-200 family (miR-200a, miR-200b, miR-200c, miR-141, and miR-429) is involved in the dynamic and reversible process of epithelial–mesenchymal transition (EMT), by means of enhancing the expression of E-cadherin and maintaining the epithelial cell phenotype [5]. EMT is a key mechanism in breast cancer progression, as it modulates cell plasticity and plays a crucial part in the invasion and dissemination of tumor cells. During early metastatic events, low levels of miR-200s promote EMT. Thereby, they enable cancer cells to acquire a mesenchymal phenotype as well as the ability to migrate and invade surrounding tissues. Conversely, in late metastatic stages, high levels of miR-200s facilitate mesenchymal–epithelial transition (MET) by promoting the expression of epithelial cell phenotypes. That process is prominently involved in promoting successful colonization in previously infiltrated organs [6, 7]. For example, related research findings reported high miR-200c levels in blood plasma of patients with advanced breast cancer [8]. Furthermore, high levels of circulating miR-200s measured in these patients’ blood samples have been found to correlate with decreased overall (OS) and progression-free survival (PFS) [9–11]. Moreover, clustered analyses, including miR-200c levels, lymph node infiltration, tumor grade, and estrogen receptor were able to predict late relapse [12]. Noticeably and enticingly, recently published findings reported that high levels of miR-200a can predict response to chemotherapy [13]. These findings imply that miRNAs may offer an invaluable opportunity to reliably predict patient outcome and/or response to specific treatment protocols that are currently being followed.

The present analysis aims to provide valuable insights related to the clinical applicability of liquid biopsies as a minimally invasive method for identifying high-risk patient populations in metastatic breast cancer. Hence, our aim is to potentially provide a platform for capturing patients in need of tailored therapeutic approaches in line with their respective overall risk for relapse/recurrence of disease parameters, with the overall goal of maintaining a higher quality of life while combating disease.

## Patients and methods

### Study design and samples

We conducted a retrospective, single-center, cohort study at the National Center for Tumor Diseases (NCT), Heidelberg, Germany, together with the German Cancer Research Center (DKFZ), Heidelberg, Germany, and the Department of Obstetrics and Gynecology, University of Heidelberg, Heidelberg, Germany. This study was approved by the Ethics Committee of the Medical Faculty Heidelberg of the Heidelberg University, approval No. S-295/2009. Written informed consent was given by all participants.

In all, 47 metastatic breast cancer patients ≥ 18 years who were about to begin with a new line of systemic therapy were consecutively enrolled between April 2010 and September 2011. Previous therapy as well as the time of initial diagnosis were disregarded.

At study entry, radiological evaluation was performed and then repeated every 3 months to classify therapy response according to RECIST criteria [14]. Survival (OS, PFS) was measured in terms of time, specifically so in months, from first blood draw after inclusion in the study and up until progression of disease, death, or loss to follow-up. Furthermore, patients were divided into two prognostic groups according to their previously established RECIST status as obtained during initial radiological assessment at the time of treatment initiation. Initially, the study set out to measure therapeutic success 3 month postinitial intervention. However, as obtaining relevant clinical markers, such as blood samples, or initiation of treatment protocol occurred with up to a week’s worth of delay, the authors found it sensible to permit assessment after 4 months in order not to unnecessarily reduce sample size and/or facilitate loss of participants. 4 month postinclusion in the study, patients displaying signs of disease progression according to radiological evaluation were grouped as such (PFS ≤ 4 months, *n* = 22) as were those with stable disease or partial/complete response (PFS > 4 months, *n* = 25). All patients were of female sex and Caucasian.

### miRNA assessment

Blood samples from patients with metastatic breast cancer were taken prior to initiating a new line of systemic treatment (baseline) and after one cycle of systemic therapy (three cycles of hormone therapy, respectively) to determine miR-200a, miR-200b, miR-200c, miR-141, and miR-429 expression levels.

### Isolation of circulating miRNAs

A total of 7.5 ml of peripheral blood was collected in 9 ml EDTA tubes (Sarstedt S-Monovette^®^, Nürnbrecht, Germany) and processed within 2 h according to a two-step centrifugation protocol: 1300* g* for 20 min at 10 °C, followed by 15,500* g* for 10 min at 10 °C. Afterwards, samples were snap-frozen and stored at − 80 °C. miRNAs were extracted from 400 µl of plasma using TRI-Reagent LS^®^ (Sigma-Aldrich, St. Louis, USA) and Qiagen miRNeasy® mini kit (Qiagen, Hilden, Germany), as described by Turchinovich et.al. [15].

All laboratory procedures were performed at room temperature (RT). First, 400 µl blood plasma was denatured in 2 ml Eppendorf tubes by adding 1,200 µl TRI-Reagent LS^®^. Then, 1 pg synthetic cel-miR-39 (spiked-in normalization reference) and 1 µl glycogen (20 mg/ml) (Thermo Scientific, Life Technologies, Carlsbad, USA) were added. Samples were vortexed for 15 s and incubated for 20 min at RT. After addition of 220 µl chloroform the samples were vortexed for 15 s, incubated for 5 min at RT, and centrifuged at 16,000* g* for 20 min. The 600 µl of upper aqueous phase containing dissolved RNA was collected, mixed with 900 µl of100% ethanol (Roth Chemicals, Karlsruhe, Germany) and incubated for 5 min at RT. Afterwards, the RNA was purified using miRNeasy^®^ mini spin columns (Qiagen, Hilden, Germany) according to the manufacturer’s instructions. Ultimately, the RNA was eluted in 60 µl RNAse-free water and stored at − 80 °C until further processing.

### Quantitative real-time PCR

Current analysis was based on analyzing a panel of 13 miRNAs developed and validated within a previously published study by Madhavan and colleagues in 2012 [9].

The analyzed miRNAs included: miR-16, miR-24, miR-29a, miR-138-5p, miR-141, miR-200a, miR-200b, miR-200c, miR-210, miR-375, miR-365, miR-429, miR-1260, as well as exogenous synthetic spike-in normalizer cel-miR-39.

In total, 5 µl of purified total RNA from 60 µl eluate was used as an input into a reverse transcription (RT) reaction performed by TaqMan^®^ MicroRNA Reverse Transcription Kit (Applied Biosystems, Carlsbad, USA). Every RT reaction having 15 µl volume in total comprised 5 µl of purified miRNA sample, 3 µl of stem-loop RT primers mix (3 per RT sample), 1.5 µl of RT buffer 10X, 0.15 µl of dNTPs 100 mM, 0.19 µl of RT inhibitor, 1 µl of MultiScribe Reverse Transcriptase 50 U/µl, and 4.19 µl RNase-free water. The RT reactions were incubated on 96-well PCR plates for 30 min at 16 °C, followed by 30 min at 42 °C, 5 min at 85 °C, and then held at 4 °C. Afterwards, RT products were diluted in RNase-free water and stored at − 20 °C.

Subsequent qPCR reactions (having final volumes of 10 µl each) included 2 µl of diluted RT product, 5 µl of TaqMan^®^ Universal PCR Master Mix (Applied Biosystems, Carlsbad, USA), 0.5 µl of corresponding miRNA assay primers and 2.5 µl RNase-free water. The qPCR reactions were incubated in LightCycler^®^ 480 384-multiwel plates (Roche Diagnostics, Mannheim, Germany) at 95 °C for 10 min, followed by 50 cycles of 95 °C for 10 s and 60 °C for 1 min. All reactions were run in duplicate. Real-time PCR was performed using LightCycler^®^ 480 Real-Time PCR System (Roche Diagnostics, Mannheim, Germany), and crossing points (Cp) were determined by the second derivative max method implemented in LightCycler^®^ 480 software. Relative quantities of miRNA were calculated using a modified ΔΔ Ct method after normalization to the cel-miR-39 spiked-in control. Specifically, the normalization factor was calculated using the difference between the mean cel-miR-39 Cp values from all samples and the mean cel-miR-39 Cp values from a respective patient sample.

### Statistical analysis

Demographic data and tumor characteristics were presented as mean, median and range for continuous variables, and in absolute and relative frequencies for categorical variables.

Boxplots displayed differences in miRNA expression levels between patient groups with early and late PFS. Student’s *t* test was performed to investigate differences in miRNA expression levels between patients with early and late progression of disease. Furthermore, Student’s *t* test was performed to assess the miRNA expression in groups with the following characteristics: chemotherapy, endocrine therapy, bone, local and visceral metastasis, respectively. Thereby, normal distribution of the miRNAs could be assumed due to the previous normalization of the miRNA values. Univariable logistic regression analysis was performed to investigate miRNA expression patterns during one cycle of systemic therapy as a predictor for early and/or late disease progression. Multivariable logistic regression analysis included the following variables: age at initial diagnosis, age at study enrollment, tumor receptor status (HR+ /HER2−, HER2+ , TNBC), chemotherapy, endocrine therapy as well as visceral, local and bone metastasis, respectively. Kaplan–Meier curves were generated alongside univariable Cox-regressions and corresponding hazard ratios with 95% confidence intervals to evaluate patient outcome. To calculate and compare survival probability, the miRNA Cp values were dichotomized into patient groups with lower and higher miRNA expression, defined as one lower quartile and three upper quartiles [9]. The level of significance was set at alpha 5%. Due to the exploratory character of this study, *p* values have to be interpreted in a descriptive sense and are not adjusted for multiplicity. No missing values have been imputed. Statistical analysis was performed using R version 3.5.1 (2018-07-02) [16].

## Results

In all, 47 consecutive patients were enrolled in this study. Patient characteristics and tumor biology at baseline are displayed in Table 1. Of the respective 47 patients, 22 suffered progression of disease within 4 months after one cycle of chemotherapy (three cycles of hormone therapy, respectively), whereas 25 experienced disease progression after more than 4 months. The median time interval between the analysis of miRNAs before starting a new systemic therapy and after one cycle of the respective therapy was 41.5 days (Range: 26–70) in patients with PFS ≤ 4 months and 40 days (26–149) in patients with PFS > 4 months, respectively.

**Table 1:** Characteristics of patients with early (PFS ≤ 4 months) and late (PFS > 4 months) relapse

Characteristic	Early (PFS ≤ 4 months)		Late (PFS > 4 months)	
	Number	Percentage	Number	Percentage
Number of patients	22	46.8	25	53.2
Age at initial diagnosis (years)				
Mean	47		52	
Range	33–72		34–77	
Age at baseline (years)				
Mean	54		60	
Range	35–89		40–47	
NST	40	45.5	11	44.0
ILC	4	18.2	4	16.0
Other	0	0	1	4.0
Unknown	8	36.4	9	36.0
HR positive / HER2 negative	9	40.9	12	48.0
HER2 positive	1	4.5	4	16.0
TNBC	4	18.2	1	4.0
Distant metastasis at initial diagnosis				
No	14	63.4	15	60.0
Yes	5	22.7	5	20.0
Unknown	3	13.6	5	20.0
Overall survival				
Median	15.6		38.2	
Range	3–54		10–84	
Progression-free survival				
Median	2.6		17.6	
Range	1–4		5–48	
Visceral metastasis				
Yes	14	63.6	11	44.0
No	8	36.4	14	56.0
Local metastasis				
Yes	9	40.1	14	56.0
No	13	59.1	11	44.0
Bone metastasis				
Yes	19	86.4	18	72.0
No	3	13.6	7	28.0
Line of therapy				
0	0	0	1	4.0
1	8	36.4	9	36.0
2	5	22.7	7	28.0
≥ 3	9	40.9	8	32.0
Chemotherapy				
Yes	21	95.5	24	96.0
No	1	4.5	1	4.0
Endocrine therapy				
Yes	16	72.7	18	72.0
No	6	27.3	7	28.0

Histology and receptor status refer to the primary tumor of the patient

*NST* invasive carcinoma of no special type, *ILC* invasive lobular carcinoma, *HR* hormone receptor, *HER2* human epidermal growth factor receptor 2, *TNBC* triple-negative breast cancer, *PFS* progression-free survival

**Fig. 1:**
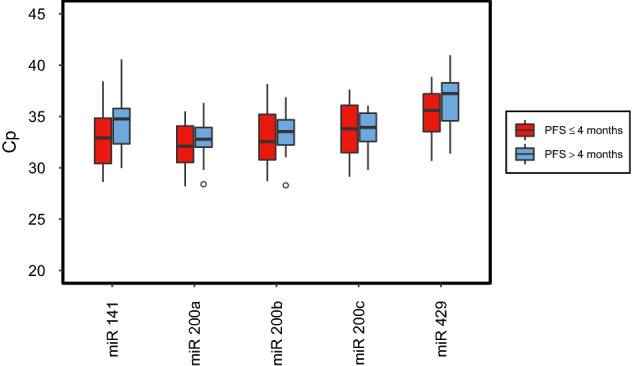
Box plots showing miRNA levels of patients with PFS ≤ 4 months compared to patients with PFS > 4 months before starting a new line of systemic therapy

**Fig. 2:**
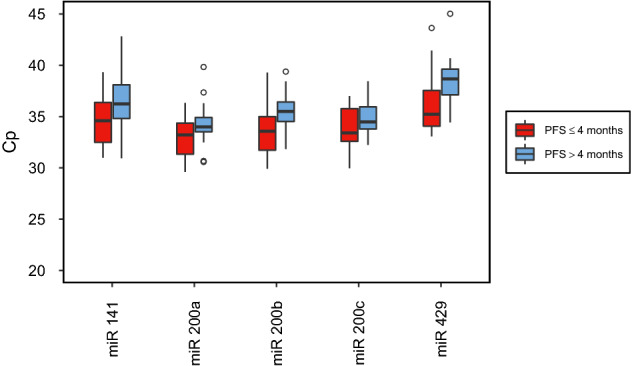
Box plots showing miRNA levels of patients with PFS ≤ 4 months compared to patients with PFS > 4 months after one cycle of a new line of systemic therapy

Figures 1 and 2 illustrate expression levels of circulating miR-200a, miR-200b, miR-200c, miR-141, and miR-429 in patient groups with either early and/or late disease progression, both before and after one cycle of systemic treatment. At both timepoints, plasma miRNA levels tended to be higher in patients with early disease progression than in patients with late disease progression. Before initiating a new line of systemic therapy, only expression of miR-429 was significantly higher in early progressive cancer (*p* = 0.024). After therapeutic intervention, however, miR-200a, miR-200b, miR-141, and miR-429 expression levels were significantly higher in patients with early progression (*p* = 0.039, *p* = 0.003, *p* = 0.017, *p* = 0.010). miR-200c was marginally not statistically significant (*p* = 0.076) (Table 2). Thereafter, univariable logistic regression analysis was utilized to analyze the aforementioned circulating miRNAs in terms of their predictive value related to PFS. Circulating miRNA levels after systemic treatment showed that miR-200a, miR-200b, miR-141, and miR-429 could predict probability of PFS. Logistic regression analysis of miR-200c and PFS, however, marginally did not reach the level of statistical significance (Table 3).

**Table 2:** Results of Student’s *t* test comparing miRNA expression in patients with early (PFS ≤ 4 months, *n* = 22) and late progression of disease (PFS > 4 months, *n* = 25)

miRNA		Baseline	After one cycle of systemic therapy
	*N*	*p*	*p*
miR-200a	47	0.246	0.039
miR-200b	45	0.438	0.003
miR-200c	47	0.860	0.076
miR-141	46	0.131	0.017
miR-429	43	0.024	0.010

*N*: sample size, *p*: statistical *p* value

**Table 3:** Results of univariate logistic regression analysis of miRNA expression after systemic therapy in patients with PFS ≤ 4 months as a predictor for early disease progression

miRNA	Estimate	*p*
miR-200a	0.337	0.048
miR-200b	0.493	0.008
miR-200c	0.290	0.076
miR-141	0.294	0.026
miR-429	0.348	0.016

Estimate: logistic regression coefficient, *p*: statistical *p* value

A potential biasing influence of systemic chemotherapy or endocrine therapy on miRNA expression was investigated by Student's *t* tests. These showed no systematic differences in miRNA expression between patients with and without chemotherapy. Only the expression levels of miR-429 in patients with endocrine therapy differed significantly after systemic therapy (*p* = 0.02). Regarding, the influence of metastatic disease, miRNA expression was different in some miRNAs in patients with local metastasis (baseline: miR-200a, miR-200b, *p* ≤ 0.05; after systemic therapy: miR-200a, miR-200c, miR-141, *p* ≤ 0.02) and bone metastasis (baseline: miR-200a, miR-200b, miR-200c, miR-141, miR-429, *p* ≤ 0.02; after systemic therapy: miR-200a, miR-200b, miR-200c, miR-141, *p* ≤ 0.002) at baseline and after systemic therapy (supplementary material, Table 1).

**Fig. 3:**
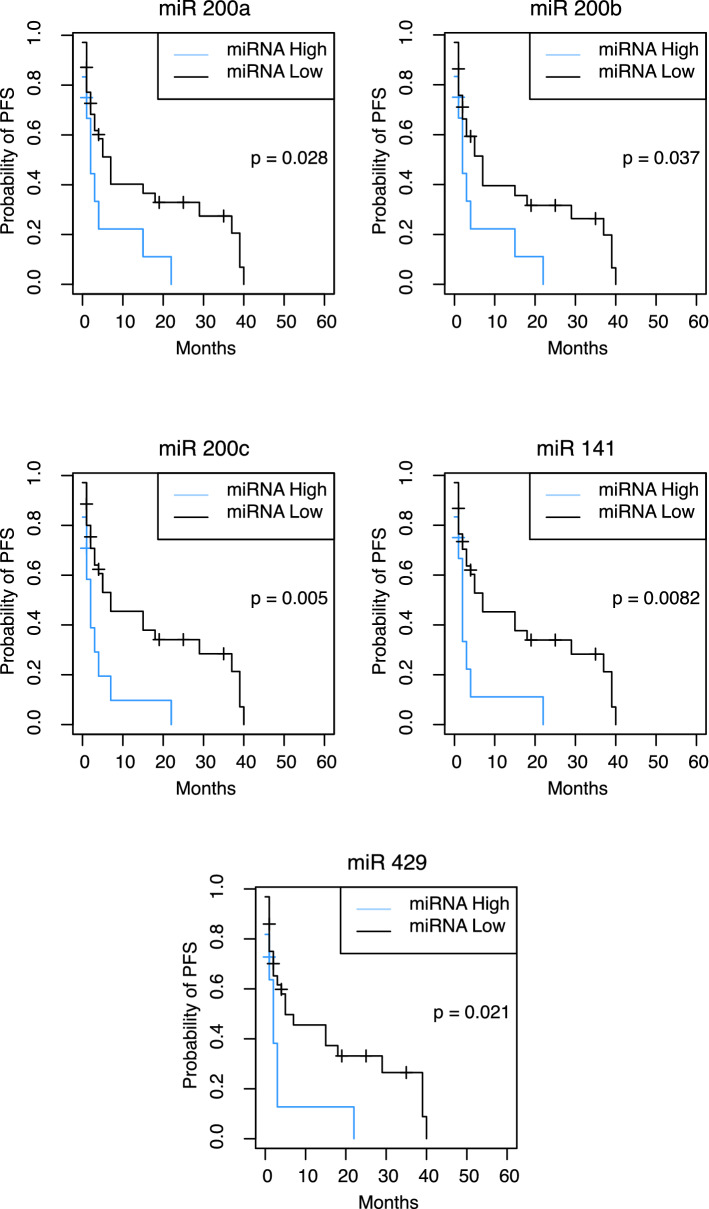
Kaplan–Meier curves of miRNA prognostic groups and progression-free survival after one cycle of systemic therapy. Sample dichotomized as lower quartile (miRNA high levels) and upper rest (miRNA low levels) based on their *Cp* values

**Fig. 4:**
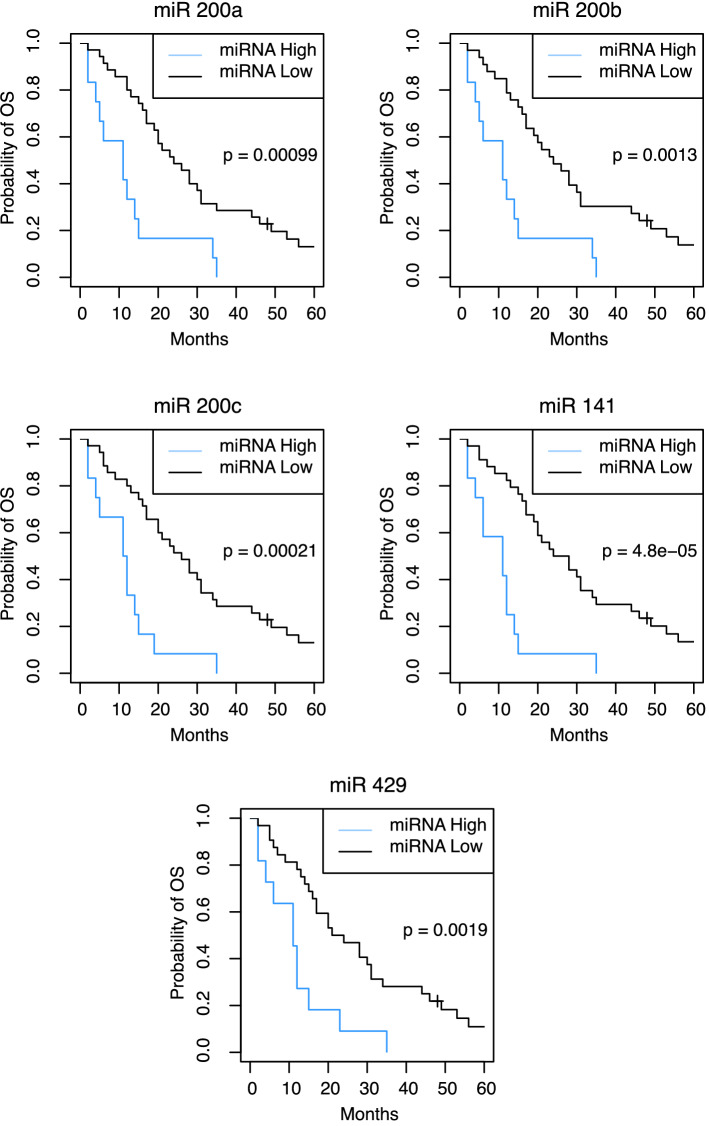
Kaplan–Meier curves of miRNA prognostic groups and overall survival after one cycle of systemic therapy. Sample dichotomized as lower quartile (miRNA high levels) and upper rest (miRNA low levels) based on their *Cp* values

In a multivariable Cox-regression model for further assessment of confounding variables, miR-200a (*p* = 0.037), miR-200b (*p* = 0.048), and miR-141 (*p* = 0.041) continued to predict early progression of disease at a statistically significant level (supplementary material, Table 2). Neither miR-200c (*p* = 0.132), miR-429 (*p* = 0.073), nor any of the potentially confounding variables accounted for in the analysis reached statistical significance, including the metastatic location. Regarding patient outcome, average PFS in patients with PFS ≤ 4 months was 2.6 (Range: 1–4) months, compared to 17.6 (5–48) months in patients with PFS > 4 months. Mean OS was 15.659 (3–54) months in patients with PFS ≤ 4 months vs. 38.2 (10–84) months with PFS > 4 months, respectively (Table 1). The comparison of survival probabilities of patients with high vs. low miRNA levels according to Kaplan–Meier curves indicated significantly lower OS and PFS among patients with heightened expression levels of miR-200a, miR-200b, miR-200c, miR-141, and miR-429, respectively (Figs. 3 and 4). Cox-regression and hazard ratios validated these results, comparing OS and PFS distribution among these prognostic groups (Table 4). Moreover, miRNAs were not only significantly related to PFS and OS as dichotomized but also as continuous variable, further strengthening their value as prognostic marker (supplementary material, Table 3).

**Table 4:** Results of univariate Cox-regression comparing OS and PFS distribution among miRNA prognostic groups after one cycle of systemic therapy

miRNA	*OS*		*PFS*	
	HR (95% CI)	*p*	HR (95% CI)	*p*
miR-200a	3.12 (1.54–6.31)	0.001	2.25 (1.05–4.82)	0.037
miR-200b	3.05 (1.49–6.22)	0.002	2.16 (1.00–4.66)	0.049
miR-200c	3.70 (1.79–7.62)	< 0.001	2.81 (1.33–5.93)	0.007
miR-141	4.27 (2.04–8.91)	< 0.001	2.74 (1.25–6.01)	0.012
miR-429	3.09 (1.47–6.47)	0.003	2.50 (1.11–5.65)	0.028

*OS* overall survival, *PFS* progression-free survival, *HR* hazard ratio, *CI* confidence interval, *p* statistical *p* value

## Discussion

This study aimed to investigate circulating EMT-specific miRNA levels in the blood of metastatic breast cancer patients during and after one cycle of systemic therapy to determine potential liquid biomarkers for monitoring both cancer progression and treatment efficacy.

Heightened expression of miR-200a, miR-200b, miR-200c, miR-141, and miR-429 is known to be associated with reduced OS and PFS probability [9, 11, 17].

The present study was able to replicate published findings by showing a significant relation between unfavorable outcome and heightened miR-200a, miR-200b, miR-200c, miR-141, and miR-429 expression. Elevated plasma levels of the aforementioned miRNAs were significantly related to worse OS and PFS after one cycle of systemic therapy. This finding is especially compelling as prognostic evaluations are of utmost importance to these patients’ risk and thus, further clinical management.

Deductively speaking, this study not only provided evidence for the prognostic ability of the miR-200 family, but could further show that these miRNAs are of predictive value for disease progression during systemic therapy and, thus, open up opportunities for clinical use as biomarkers in breast cancer management.

This analysis did not show any significant differences in miRNA expression levels among patients with early (PFS ≤ 4 months) and late (PFS > 4 months) PFS before starting a new line of systemic therapy. As patients were affected by metastatic progression at the time of study enrollment, which has been shown to be related to heightened miR-200s levels, it makes sense that miRNA expression patterns do not differ significantly between study groups [9, 11, 18, 19]. In addition, differences in miRNA expression levels were observed in patients with local and bone metastases. It is crucial to note that the discordances were already observed at baseline and also seen after one cycle of therapy. A confounding capacity of said variables on the predictive value of miRNA on an early progression after therapy could not be confirmed by subsequent regression analysis. Remarkably, however, this study proved that after therapeutic intervention, the expression of 4 (miR-200a, miR-200b, miR-141, and miR-429) out of 5 members of the miR-200 family was significantly different in patients who suffered progression of disease within 4 months or less than in those who did not. In addition, these 4 miRNAs were found to be significant predictors of early relapse after therapeutic intervention. In line with these findings, other research showed a correlation of miR-141, miR-200a, and miR-429 with Stage IV breast cancer and, even more interesting, suggested that high miR-141 serum expression is associated with shorter disease-free survival in metastatic cancer of the brain as well as a predictive value for decreased PFS [10, 20]. Furthermore, circulating miR-200c was recently shown to distinguish relapsed from non-relapsed patients with early breast cancer and, combined with lymph node infiltration, estrogen receptor status, and tumor grade, even to predict occurrence of late relapse [12]. Supporting the miR-200 cluster as clinically valuable biomarkers, another study reported elevated serum levels of miR-200a as predictive of resistance to chemotherapy in metastatic breast cancer patients, regardless of treatment regimen [13]. Moreover, Madhavan et al. reported that miR-200a, miR-200b, and miR-200c expression was predictive of metastatic onset, as upregulated plasma levels were measured 2 years before clinical diagnosis of breast cancer metastasis [21].

Since prognostic evaluations are of utmost importance for the further clinical management of these patients in terms of individualized treatment plans and follow-up for high-risk patients, our findings are especially intriguing.

Although miR-200c did not quite reach the level of significance in terms of alpha 5%, it is important not to neglect its involvement in the pathomechanisms of breast cancer, especially with regard to its genetic location. In humans, the miR-200 family is located at two sites of the genome: miR-200a, miR-200b, and miR-141 form a cluster on chromosome 1, and miR-200c and miR-141 build a cluster on chromosome 12, respectively. Acknowledging, that miR-141 and miR-200c share the same genetic heritage and exert collective roles in the metastatic process, their respective, similar *p* values are reasonable (*p*_miR-141_ = 0.026, *p*_miR-200c_ = 0.076) [17, 22]. In favor of this hypothesis, Dyxhoorn et al. reported that the miR-141-200c cluster facilitates post extravasation events in lung cancer metastasis [6]. In addition, another study reported an association of this genetic location with biliary tract cancer [23]. When discussing and interpreting results based solely on *p* values defined by dichotomous cutoff values, it is important not to overlook information that might possibly strengthen previous findings, such as the dual role of miR-141 and miR-200c in regulating metastasis [24]. It must also be borne in mind that the small sample size may have had an impact on the results. Taking these considerations into account, this study suggests that miR-200c and its predictive relation to PFS should not be disregarded.

Due to their ability to identify high-risk patients, miR-200a, miR-200b, miR-141, and miR-429 are promising markers in the clinical setting for determining which patients in fact require and benefit from further, more intensive adjuvant therapeutic interventions as opposed to those who would be treated unnecessarily.

Given the still sparse data on the clinical use of miR-200s in metastatic breast cancer, this study further aims to improve the basic understanding of the dynamics of their expression patterns under therapy to contribute knowledge for a potential clinical application. Exemplarily, ongoing research showed that elevated miR-200 family levels correlate with decreased expression of the immune checkpoint protein PD-L1, thereby hypothesizing that measuring miR-200 levels might help identify patients with PD-L1 expression who would fit a respective therapy [25]. In addition, by interfering with the immune checkpoint protein expression, the miR-200 family might not only serve as a predictive marker but further be a potential therapeutic target and/or agent. [25–28]

Concluding, the presented results are of utmost clinical importance and need to be validated by further comprehensive studies, because as a predictive marker capable of identifying patients at high risk of disease progression, the miR-200 family has the potential to profoundly impact and improve clinical decision-making processes, thereby improving overall patient outcome and quality of life.

## Limitations

Limiting factors of this study are the small sample size, which may have influenced the results and must be considered when interpreting the results. Unfortunately, due to the restricted number of cases and partly missing histological data, a valid statement about the association and expression of miR-200 expression in different tumor subtypes of this study collective is not possible. Regarding miRNA extraction, there is a lack of a universally accepted normalization method [29]. Therefore, *Cp* values of miRNA expression measured in plasma are subject to the limits of the quality of the respective normalization strategy. Finally, it should also be noted that *Cp* values above 35 may indicate inefficient miRNA extraction and should, therefore, be interpreted carefully.

## Conclusions

Circulating miRNAs are differentially expressed in plasma of patients with late and/or early relapse. Four out of 5 members of the miR-200 family circulating in plasma predicted progression-free survival during systemic therapy. The miR-200 family is a valuable prognostic marker for overall and progression-free survival.

## Supplementary Information

Below is the link to the electronic supplementary material.

Supplementary file1 (DOCX 18 KB)

## Data Availability

The data sets generated during and/or analysed during the current study are available from the corresponding author on reasonable request.
