# Supplementary material for: Circulating miR-200 family as predictive markers during systemic therapy of metastatic breast cancer
Source: Arch Gynecol Obstet. 2022 Mar 2;306(3):875–85. doi: 10.1007/s00404-022-06442-2 (PMC9411224; doi:10.1007/s00404-022-06442-2)
Supplement: Supplementary file 1 — Supplementary file1 (DOCX 18 KB) [file 404_2022_6442_MOESM1_ESM.docx]

**Supplementary Material**

**Table 1** Results of Student's t-test of miRNA expression levels in patients with and without chemotherapy, endocrine therapy, visceral metastasis, local metastasis and bone metastasis, respectively, at baseline and after one cycle of systemic therapy.

|  | *Chemotherapy, p* | | *Endocrine Therapy, p* | | *Visceral MT, p* | | *Local MT, p* | | *Bone MT, p* | | |  |
| --- | --- | --- | --- | --- | --- | --- | --- | --- | --- | --- | --- | --- |
| *miRNA* | *Baseline* | *After systemic therapy* | *Baseline* | *After systemic therapy* | *Baseline* | *After systemic therapy* | *Baseline* | *After systemic therapy* | *Baseline* | *After systemic therapy* | |  |
| miR-200a | 0.97 | 0.79 | 0.54 | 0.50 | 0.84 | 0.76 | 0.03 | 0.002 | 0.001 | 0.001 | |  |
| miR-200b | 0.94 | 0.45 | 0.92 | 0.79 | 0.52 | 0.87 | 0.05 | 0.09 | 0.004 | 0.001 | |  |
| miR-200c | 0.82 | 0.80 | 0.45 | 0.90 | 0.71 | 0.69 | 0.09 | 0.01 | 0.02 | 0.002 | |  |
| miR-141 | 0.86 | 0.45 | 0.80 | 0.97 | 0.31 | 0.57 | 0.15 | 0.02 | 0.001 | 0.001 | |  |
| miR-429 | 0.98 | 0.55 | 0.78 | 0.02 | 0.55 | 0.89 | 0.08 | 0.12 | 0.01 | 0.23 | |  |
| Note. MT- metastasis, *p* - statistical p-value | | | | | | | | | | |  | |

**Table 2** Results of multivariable logistic regression analysis of miRNA expression after systemic therapy in patients with PFS ≤4 months as a predictor for early disease progression.

|  | *miR-200a* | | *miR-200b* | | *miR-200c* | | *miR-141* | | *miR-429* | |
| --- | --- | --- | --- | --- | --- | --- | --- | --- | --- | --- |
|  | Estimate | *p* | Estimate | *p* | Estimate | *p* | Estimate | *p* | Estimate | *p* |
| miRNA | 0.500 | 0.037 | 0.456 | 0.048 | 0.365 | 0.054 | 0.367 | 0.041 | 0.284 | 0.073 |
| Age at initial diagnosis | -0.118 | 0.211 | -0.047 | 0.652 | -0.101 | 0.263 | -0.093 | 0.318 | 0.043 | 0.689 |
| Age at baseline | 0.137 | 0.128 | 0.079 | 0.382 | 0.105 | 0.189 | 0.121 | 0.148 | 0.006 | 0.943 |
| HR positive/ HER2 negative | 2.345 | 0.067 | 1.069 | 0.378 | 1.207 | 0.228 | 1.128 | 0.288 | 0.496 | 0.659 |
| HER2 positive | 2.847 | 0.097 | 1.573 | 0.343 | 2.288 | 0.128 | 1.725 | 0.273 | 1.552 | 0.352 |
| TNBC | -2.347 | 0.235 | -2.176 | 0.352 | -1.998 | 0.293 | -1.469 | 0.434 | -1.388 | 0.495 |
| Local Metastasis | -0.347 | 0.717 | -0.414 | 0.510 | -0.134 | 0.808 | -0.316 | 0.605 | -0.179 | 0.753 |
| Visceral Metastasis | -0.803 | 0.337 | 0.649 | 0.573 | 0.090 | 0.932 | 0.329 | 0.764 | 0.952 | 0.422 |
| Bone Metastasis | -1.019 | 0.412 | 0.768 | 0.574 | 0.068 | 0.953 | 0.726 | 0.569 | -0.301 | 0.800 |
| Chemotherapy | -0.665 | 0.723 | -0.434 | 0.755 | -1.119 | 0.394 | 0.061 | 0.968 | -0.221 | 0.879 |
| Endocrine therapy | -1.807 | 0.165 | -0.170 | 0.598 | -0.170 | 0.558 | -0.216 | 0.446 | -0.126 | 0.670 |
| Note. PFS - progression-free survival, Estimate - logistic regression coefficient, HR - hormone receptor, HER2 - growth hormone receptor 2, TNBC - triple negative breast cancer, *p* - statistical p-value | | | | | | | | | | |

**Table 3** Results of univariable Cox-regressions comparing OS and PFS distribution among miRNA as continuous variables after one cycle of systemic therapy

|  | *OS* | |  | *PFS* | |
| --- | --- | --- | --- | --- | --- |
| *miRNA* | HR (95% CI) | *p* |  | HR (95% CI) | *p* |
| miR-200a | 1.24 (1.03-1.48) | 0.020 |  | 1.24 (1.03-1.49) | 0.023 |
| miR-200b | 1.31 (1.10-1.58) | 0.003 |  | 1.27 (1.05-1.54) | 0.013 |
| miR-200c | 1.27 (1.05-1.55) | 0.016 |  | 1.25 (1.01-1.56) | 0.044 |
| miR-141 | 1.25 (1.10-1.41) | <0.001 |  | 1.15 (1.02-1.31) | 0.028 |
| miR-429 | 1.25 (1.07-1.45) | 0.004 |  | 1.17 (0.99-1.38) | 0.056 |
| Note. OS - overall survival, PFS - progression-free survival, HR - hazard ratio, CI - confidence interval, *p* - statistical p-value | | | | | |
